# Relationship between Musculoskeletal Disorders and Work Performance of Nursing Staff: A Comparison of Hospital Nursing Departments

**DOI:** 10.3390/ijerph18137085

**Published:** 2021-07-02

**Authors:** Yang-Kun Ou, Yi Liu, Yu-Ping Chang, Bih-O Lee

**Affiliations:** 1 Department of Creative Product Design, Southern Taiwan University of Science and Technology, Tainan 710301, Taiwan; ouyk@stust.edu.tw; 2 Department of Medical Research, School of Nursing, Kaohsiung Medical University Hospital, Kaohsiung 807378, Taiwan; gn94yliu@kmu.edu.tw; 3 School of Nursing, The State University of New York, University at Buffalo, New York, NY 14214-3079, USA; yc73@buffalo.edu

**Keywords:** nursing, characteristics of workplace, prevalence rate, odds ratio, ergonomics

## Abstract

This study aimed to explore the relationship between musculoskeletal disorders and the work performance of nursing staff. This cross-sectional study used the Checklist for Musculoskeletal Disorders (MSDs), the Nordic Musculoskeletal Questionnaire (NMQ), and the Work Ability Index to survey the prevalence of MSDs, as well as to determine the impact of MSDs on 117 nursing staff working in an emergency department, an intensive care unit, and general wards. The findings indicate that the nursing staff were exposed to a workplace environment with a high risk of MSDs. Nursing staff in the emergency department were at a particularly high risk of MSDs in their upper and lower limbs, while those working in the intensive care unit had a particularly high risk of injuries caused by manual material handling. Analyzing the relationship between MSD risk factors and NMQ scores showed a 6 times, 3.25 times, and 2.28 times increase in MSD conditions with a high workload compared to a low workload in the hand and wrist, the lower back or waist, and the knee, respectively. Medium and high workloads were found to increase the risk of MSD, which in turn affected the work ability of the nurses.

## 1. Introduction

The primary functions of the musculoskeletal system include enabling motion, offering protection, supporting the body, and maintaining body homeostasis. Overexertion, fatigue, prolonged loads, insufficient oxygen, and repetitive activities can reduce muscle contraction [1]. Lack of rest may induce injury risks. Musculoskeletal disorders (MSDs) involve pain and inflammation in body tissues (e.g., muscles, tendons, and nerves), reduced motor function, or muscle/bone discomfort caused by the continuous exertion of force and repeated movements [2]. Generally, MSDs are soft tissue inflammation in the body or degenerative diseases such as tendinitis, muscle strain, joint degeneration, nerve compression, or tenosynovitis. Symptoms of MSDs include pain, soreness, swelling, and restriction of posture angle. In addition to acute trauma, MSDs are mostly caused by chronic injuries attributable to long-term poor posture, repetitive movements, improper force exertion, and overloading [3].

Long-term clinical work subjects nurses to occupational risks for MSDs. The Occupational Hazards Survey conducted in the United States in 1984 revealed that the medical service industry ranked fourth in terms of occupational injury and illness incidence rate. Nursing staff accounted for 10.9% of the reported cases. Usually occupying two-fifths to one-half of total hospital staff, nurses are directly exposed to occupational hazards and risks (e.g., needle-stick injuries, violence, chemical exposure, over-exertion, and work-related stress) when delivering first-line clinical care. Prolonged standing and repetitive tasks such as handling/carrying objects, assisting with patient handling, changing patient wound dressings, injections, and turning patients over in bed render nurses as a group at high risk of MSDs [4,5,6,7,8]. Nursing staff are occupied with complicated work tasks and must handle various clinical tasks under time constraints and considerable stress. The literature indicates that the medical institution level, workload, job satisfaction, working hours, work-related stress, organizational climate and support, physical load of individuals, job tenure, gender, and exercise habits all affect the incidence rate of MSDs, which mostly occur in the shoulders, neck, or lower back [9,10,11,12,13,14,15,16,17,18]. Although not fatal, MSDs are likely to reduce work efficiency and quality of life because of the long disease course and become a factor in high turnover rates among nursing staff [7,19].

Nursing staff spend approximately 20% of their working time handling and moving patients [20]. According to the results of a survey conducted by the American Nurses Association in 2011, 62% of the nursing staff surveyed were highly concerned about MSDs, and 56% of the nursing staff developed MSDs or experienced worsened injuries or illnesses from work [21]. In Taiwan, only a few published studies have investigated MSDs among nurses, and were either conducted as a population-based study using diagnostic codes from the National Health Insurance Research Database [22], or surveys using self-report questionnaires without complementary objective evaluations [23,24]. Therefore, this study aims to improve understanding of the settings that contribute to MSDs in nurses. The objectives of this study were: (1) to survey work risk factors for MSDs in nurses using the ergonomic-based checklist for MSDs (hereafter, the “Checklist for MSDs”), explore MSD prevalence and work ability using the self-reported Nordic Musculoskeletal Questionnaire (NMQ) and Work Ability Index; and (2) to analyze relationship between MSD risk factors, MSD conditions, and work ability among nurses from different departments in the hospital.

## 2. Materials and Methods

### 2.1. Research Design

Based on a cross-sectional design, this study explored the relationship between the Checklist for MSDs and the Work Ability Index of nursing staff.

### 2.2. Participants and Setting

We recruited nursing staff from a teaching hospital in southern Taiwan with the following inclusion criteria: nursing staff with at least 3 years of seniority and aged no older than 45 years. Those who had painful sequelae from a traumatic injury (e.g., automobile accident or fall) or were pregnant were excluded. After an explanation of the study objectives by the researchers, the participants were asked to sign a consent form and complete the questionnaire. Subjects were recruited from each department by verbal invitation and joined the study on a voluntary basis, and stratified sampling was used based on the number of different units, such as the emergency department, the intensive care unit, and general wards. The general wards in this particular hospital are mostly admissions requiring medical-surgical care.

### 2.3. Research Data Collection

The research design for this study was approved by the Institutional Review Board of Chang Gung Medical Foundation (IRB No.: 201700922B0; date of approval: 25 July 2017). Data collection was conducted after obtaining the consent of the clinical ethics committee. Before data collection, an explanation was provided to the nursing departments of the hospital, and a trained, experienced research assistant was assigned to each nursing department to observe, evaluate, and interview nursing staff.

### 2.4. Research Tools

A scale-based assessment questionnaire was used to collect data for analysis, which included demographic data, the Checklist for MSDs for objective assessment of workload during work, the NMQ to characterize MSD symptoms, and the Work Ability Index to explore the effects of MSDs on work. All the surveys were paper-and-pencil.

#### 2.4.1. Demographic Data

The demographic data were gender, age, height, weight, corresponding department, pregnancy history (for women only), frequency of exercise, tobacco use, and dominant hand.

#### 2.4.2. The Checklist for MSDs

This checklist was designed by the United States Occupational Safety and Health Administration with a focus on identifying combinations of the most common and influential risk factors contributing to MSD [25]. The Chinese version of the checklist promoted by Taiwan’s Institute of Occupational Safety and Health (IOSH) is part of a government publication (in Chinese) freely available on the internet [26]. When evaluating across various occupations, the Checklist for MSDs was found to have an overall sensitivity of 0.537 and a specificity of 0.654 [27]. This observation checklist comprises two main scales. Scale I assess the risk factors of the upper limbs, and Scale II assesses the risk factors of the back and the lower limbs. There is also a Scale III, which is considered a part of Scale II. Scale III assesses risk factors in manual material handling, and its total score should be combined with Scale II’s score in calculation.

Scale I covers seven major risk factors, namely repetitive tasks (finger, wrist, elbow, and shoulder or neck movements), hand exertion (repetitive tasks or static loads), poor posture, contact pressure, vibration, environment, and control of working speed. Scale II consists of five major risk factors, namely poor posture (a repetitive posture or static posture), contact pressure, vibration, push/pull, and control of working speed. Scale III assesses the risk factors of manual material handling, such as weight, position, frequency, posture, walking distance, and duration. Each risk factor in the checklist has its own scoring standard; the score increases with working hours.

To determine the risk factor of each participant, the risk factor items in Sections A and B of Scales I and II were cross-referenced through observation during the study. The risk factor items were placed in respective sections corresponding to working hours. Sections C, D, and E, respectively, refer to 2–4 working hours, 4–8 working hours, and >8 working hours (with 0.5 added for each additional hour). The scores of Sections C–E were summarized in Section F. Scale III’s total score was combined into Scale II’s score in the calculation. Regardless of the scale, the higher the score, the more dangerous the job. If an individual receives a total score of >5 points on either Scale I or Scale II, their job is considered to be high risk for MSDs.

The Checklist for MSDs is usually administered by a trained observer who follows along throughout the subject’s workday and evaluates the latter’s hazards for occupational injuries while doing work. For this study, the Checklist for MSDs was performed by a research assistant observing the subject’s entire workday, except for breaks, to rate the subject’s different postures during work against the checklist. Photos of the different postures for work were also taken during the observation.

#### 2.4.3. Nordic Musculoskeletal Questionnaire (NMQ)

Developed by a team of northern European researchers, this standardized questionnaire assesses work-related musculoskeletal symptoms and injuries common among operators in a workplace. Based on the subjective opinions of participants, this questionnaire investigates nine common work-related musculoskeletal symptom sites, namely the neck, shoulders, upper back, lower back or waist, elbows, hands or wrists, hips or thighs, knees, and ankles or feet. This questionnaire has been widely and internationally applied for the classification and investigation of occupational injuries, with a reliability ranging from 77% to 100% and a validity ranging from 80% to 100% [28]. The Chinese version of the NMQ commonly used in Taiwan was published as a part of MSD prevention guidelines [29], its content validity index (CVI) was found to be 0.85 as assessed by specialists in nursing, public health, ergonomics, statistics, and physicians, while its coefficient of correlation was 0.92 for work status and 0.93 for musculoskeletal disorders [30].

#### 2.4.4. Work Ability Index

Developed by the Finnish Institute of Occupational Health in 1980, the Work Ability Index evaluates individual work competency, future job requirements, and physical and psychological work performance. This index can also be used to monitor individuals or groups. Translated by the Ministry of Labor in 2010 with a retest reliability of 0.81, construct validity of 0.80, and Cronbach’s alpha of 0.74 [31], the Chinese version of the Work Ability Index used in this study covers the following seven areas: (1) current work ability compared with one’s lifetime best; (2) work ability in relation to the demands of the job; (3) number of current diseases and injuries diagnosed by a physician; (4) estimated work impairment due to disease and injury; (5) amount of sick leave taken during the previous 12 months; (6) self-prognosis of work ability 2 years into the future; and (7) mental resources.

### 2.5. Statistical Analysis

Variance in the results was analyzed using SPSS v22.0 statistical software (IBM Corp., New York, NY, USA), and post hoc analyses were conducted using the least significant differences (LSD) test. The data were expressed as mean ± standard error (SE). Categorical variables were analyzed using the chi-square test. In the logistic regression model was used to estimate the Checklist for MSDs, NMQ, and Work Ability Index on the probability of hazard ratio. The level of significance used for all analyses was α < 0.05.

## 3. Results

### 3.1. Demographic Data

A total of 117 nurses from the emergency department, intensive care unit, and general wards participated in the study. According to the demographic data (Table 1), a wide gender gap existed: 115 (98.3%) of the participants were women, and 2 (1.7%) were men. In terms of pregnancy history, 33 (28.2%) and 84 (71.8%) of the participants had and had not experienced pregnancy, respectively. The participants in the emergency department and the intensive care unit had the lowest (30.7 years) and highest (31.2 years) mean age, respectively. Regarding frequency of exercise, 47 (40%) and 10 (8.3%) of the staff rarely exercised and exercised at least once a week, respectively. One (0.8%) nurse in the intensive care unit smoked tobacco. Finally, most (96.7%) of the participants were right-handed.

### 3.2. The Checklist for MSDs

#### 3.2.1. Risk Factors for Upper Limbs

We discovered that all 117 subjects had a score over 5 in either Scale I or Scale II of the Checklist for MSDs, suggesting that they are all at a high risk of MSDs. Analysis of variance (ANOVA) showed a significant difference between the three departments for risk in the upper limbs (F(2.114) = 6.31, *p* =  0.003). The post hoc test showed a higher proportion with high risk in the emergency department (22.32) and in general wards (21.85) than those working in the intensive care unit (20.24). Observation revealed that nursing staff frequently switched between standing and sitting positions while working. Because the computer stand or mobile medical cart failed to match the height of each staff member, nursing staff had to lean forward to review the information displayed on the screen, which caused them to remain in a poor posture for long periods.

#### 3.2.2. Risk Factors for Lower Limbs

According to the ANOVA results, significant differences were present in the risk factors for lower limbs among participants in different departments (F(2.114) = 32.22, *p* < 0.001). The post hoc test showed that the highest risk factor score was observed for the participants in the emergency department (17.36), followed by those in general wards (12.51) and those in the intensive care unit (10.89). Through observation, we discovered that nursing staff had to exert great force to push and pull wheelchairs and hospital beds, step on a pedal to switch between fixed and mobile modes of hospital beds, and lean forward and bend sideways when carrying patients to the wheelchair or hospital bed or helping them transfer. The nursing staff in the emergency department and general wards were constantly standing and walking. Therefore, they both received a mean score of 0.6 on postures without leg support, which was much higher than the score yielded by the nursing staff from the intensive care unit. In addition, the nursing staff in the emergency department received a mean score on kneeling and squatting postures that was much higher than those received by the nursing staff in the intensive care unit and general wards.

#### 3.2.3. Risk Factors for Manual Material Handling

According to the ANOVA results, the participants differed significantly by department in manual material handling risks (F(2.114) = 50.45, *p* < 0.001). The post hoc test showed that the participants in the intensive care unit (9.91) were exposed to much greater manual material handling risks than the participants in the emergency department (5.64) and general wards (5.98) were. Through observation, we discovered that patients in the intensive care unit were confined to bed all day, where they received more rigorous and comprehensive care. To prevent the occurrence of pressure ulcers caused by prolonged bed rest, nursing staff conducted patient turning and repositioning several times a day, as well as giving sponge baths. The nursing staff in the emergency department and general wards did not or rarely performed the aforementioned tasks because most of their patients were able to move on their own. This explained why the participants in the intensive care unit received higher scores in manual material handling risks.

### 3.3. Nordic Musculoskeletal Questionnaire (NMQ)

Table 2 presents the MSD prevalence for each body part in the previous year. A higher prevalence of MSD was found in the neck, shoulders, and lower back/waist than in other parts of the body in nurses working in the emergency department (χ^2^ (8) = 58.14, *p* < 0.001), the intensive care unit (χ^2^ (8) = 86.45, *p* < 0.001), and the general wards (χ^2^ (8) = 93.44, *p* < 0.001).

In terms of the “duration of symptoms” for the subjects, 38 (82.7%) nurses working in the intensive care unit reported shoulder discomfort lasting for one month, which is a significantly higher proportion than those working in general wards and in the emergency department (χ^2^ (2) = 21.16, *p* < 0.001); for shoulder discomfort lasting over three years, the proportion in nurses working in the intensive care unit (45.7%) and emergency department (52%) was higher (χ^2^ (2) = 8.41, *p* = 0.02) than those working in general wards; for wrist discomfort lasting for one month, the proportion in nurses working in the intensive care unit (58.7%) was higher (χ^2^ (2) = 12.83, *p* = 0.01) (Table A1 in Appendix A) than those working in the other two departments—these findings suggest that working in the intensive care unit is associated with discomfort in the shoulders and the wrists. In terms of the subjects feeling a “presentation of pain in specific body parts”, the most common presentation was soreness, followed by numbness, then stabbing pain, occurring mostly in the neck, shoulders, upper back, lower back, or waist. Despite there being 10–15 subjects who reported soreness, there was no significant difference between the departments for soreness in the wrists, hips, thighs, knees, and ankles (Table A1 in Appendix A). In terms of the feeling of “affecting work and daily life”, most nurses expressed a slight decrease in the ability to do work. Working ability affected by shoulder symptoms was higher in those working in the intensive care unit and general wards compared to those in the emergency department (χ^2^ (2) = 14.00, *p* = 0.001), and one nurse working in the intensive care unit had lower back symptoms that prevented her ability to work. Nurses working in the intensive care unit and general wards who spend a long time standing and stepping on foot pedals to fix or move hospital beds were found to have ankle symptoms compromising their ability to work more than those working in the emergency department (χ^2^ (2) = 14.57, *p* = 0.001) (Table A1 in Appendix A).

### 3.4. Work Ability Index Questionnaire

According to the results of the ANOVA test, significant differences in work ability were observed between participants in different departments (F(2.114) = 4.496, *p* = 0.013), and the total scores of the Work Ability Index received for the emergency department (31.68) and the intensive care unit (32.48) were significantly lower than the score received for the general wards (34.07), indicating that the nursing staff in the general wards exhibited a greater work ability.

### 3.5. Hazard Ratio between the Checklist for MSDs and the NMQ

The objective Checklist for MSDs and the subjective NMQ were analyzed to explore the relationship between the workload and the MSDs of nursing staff by using a hazard ratio analysis. The Checklist for MSDs comprises three scales, namely Scales I, II, and III, for assessing the workload of body parts. This workload was analyzed based on the Checklist for MSDs and corresponding body parts in the NMQ.

The objective Checklist for MSDs and the subjective NMQ were analyzed. As Scale I is a checklist of risk factors for the upper limbs, only the two hand-related body parts covered by the NMQ (i.e., “elbows” and “hands and wrists”) were analyzed. Based on the scoring system of Scale I of the Checklist for MSDs, the workload was divided into three levels: low (<19), medium (19–24), and high (>24). The results indicated that medium and high workloads, respectively, induced a 5.75- and a 6-times increase of risk of MSDs in the hands and wrists of the nursing staff (Table 3).

Scale II of the Checklist for MSDs is a checklist of risk factors for the back and lower limbs, and five lower extremity body parts are covered by the NMQ (i.e., upper back, lower back or waist, hips or thighs, knees, and ankles or feet). Based on the scoring system of Scale II of the Checklist for MSDs, the workload was divided into three levels: low (<10), medium (10–14), and high (>14). The results indicated that medium and high workloads, respectively, induced a 1.86- and a 3.25-times higher risk of MSDs in the lower back or waist of the nursing staff, and that medium and high workloads, respectively, induced a 1.75- and 2.28-times higher risk of MSDs in the knees of the nursing staff (Table 4).

Because Scale III of the Checklist for MSDs is a checklist of risk factors for manual material handling, it subdivides the movements of various body parts during handling. Analysis was conducted of movements and the body parts performing these movements. For some movements, all the participants, regardless of department, were categorized into the same group (i.e., either all or none of them performed such movements). Therefore, only three movements that exhibited interdepartmental differences were analyzed, namely rotating the body while handling materials, material handling with one hand, and walking more than 3 m while handling/carrying materials. Because rotating the body while handling materials mainly puts a load on the lower back and waist, this movement was analyzed. Because material handling with one hand mainly puts a load on elbows, hands, and wrists, and because walking more than 3 m while handling/carrying materials requires whole body movement, all body parts covered by the NMQ were analyzed. The results are shown in Table 5. The increase in risk of MSDs in participants who walked more than 3 m while handling/carrying materials was 3.16 times in the neck, 2.03 times in the shoulders, 1.6 times in the elbows, and 1.6 times in the lower back or waist.

### 3.6. Correlation between the NMQ and the Work Ability Index

In this study, the NMQ was divided into upper limb and lower limb scales. Survey results for questionnaire items such as time of symptom onset, duration of symptom, the symptom’s impact on work and life, and frequency of symptom occurrence were used for correlation analysis with the Work Ability Index, thereby exploring the relationship between MSDs and the Work Ability Index. In the upper limb scale of the subjective NMQ, most items were significantly correlated with the Work Ability Index, except for frequency of symptom occurrence, which was non-significantly correlated with the Work Ability Index (Table 6). In the lower limb scale of the subjective NMQ, all variables were significantly correlated with the Work Ability Index (Table 6).

## 4. Discussion

In this study, the results of the MSD survey indicate that the nursing staff were a high-risk population for MSDs, and their working environments put them at high risk of MSDs. Specifically, the nursing staff from the emergency department were exposed to higher risks of MSDs than were the intensive care unit and general wards nursing staff. This finding differs from results reported elsewhere [32,33,34], where intensive care unit nursing staff had higher risks of injuries and illnesses than did nursing staff in other departments. Most MSDs were observed in the neck, shoulders, and lower back or waist of the nursing staff, and the most common type of pain induced by MSDs was soreness, followed by numbness—these findings are consistent with other studies in terms of body parts and symptoms [13,35,36,37,38]. Regardless of department, the majority of the nursing staff experienced varying levels of MSD-induced impact on their work and life, which concurs with the findings of relevant studies [35,39], as well as more recent surveys in Brazil [40] and the Czech Republic [41], and a large-scale survey in China [42]. As a possible predisposing factor, many nursing professionals were found to do inadequate exercise and to have pre-existing musculoskeletal problems in the lower back and neck even before they entered the workforce [43]. Causes of discomfort include long-term poor posture, stress from work without sufficient rest, handling/transferring patients, improper design of treatment carts/work carts/chairs, failure to use suitable tools, and wearing inappropriate work shoes without insoles [44,45,46,47,48,49], all of which are consistent with our on-site observations.

While other investigations, such as Akodu and Ashalejo [50], into the relationship between work ability and work-related musculoskeletal disorders assessed by the NMQ did not find a significant association, our study explored the relationship by examining time of symptom onset, duration of symptom, degree of impact of symptom, as well as frequency of symptom occurrence separately by upper and lower limbs and found significant correlation in all but frequency of symptom occurrence in the upper limbs. For the investigation of work ability, all subjects responded negatively regarding their performance with respect to physical and psychological demands and their future, which concurs with the finding of Amick et al. (1998) [51]. This may be attributable to the high probability of physical fatigue under immense stress and high physical demands [52].

This is one of the very few studies on nurses using human factor scales such as the Checklist for MSDs, and a lot of effort was made to collect data on all 117 subjects, as both self-reported and observational surveys (hence, both subjective and objective assessments) on musculoskeletal conditions were conducted for every subject. This study found that nursing staff in the emergency department were at a high risk of MSDs in their upper and lower limbs, while those working in the intensive care unit had a high risk of injuries caused by manual material handling. This suggests that the hospitals and nursing managers should pay close attention to those risk factors. Furthermore, they need to regularly assess the musculoskeletal condition of the nursing staff and focus on different body parts based on different departments. In addition, on-the-job training for nurses may need to incorporate the findings from this study to keep nurses aware of those injuries brought on by their daily work.

The limitations of this study are the small sample size due to the time- and effort-consuming nature of human factors scales such as the Checklist for MSDs, and no comparisons were made between male and female nurses. Possible ways to shorten the assessment time for the Checklist for MSDs include developing artificial intelligence (AI) measurement tools for MSD risk factors during work. Future studies could also explore the differences between male and female nurses. Recommended measures to improve MSDs among nurses include, but are not limited to, continuing education on ergonomic hazard prevention, the development and use of assistive devices as well as incentives for their use, and even changes in work scheduling to allow for adequate resting between work shifts.

## 5. Conclusions

This study provided evidence on the prevalence of MSDs by specific body parts in nursing staff working in different hospital departments. Through self-reported (subjective) surveys and objective observation, we described the patterns of MSDs among the nursing staff of a teaching hospital in southern Taiwan, and explored the hazards of poor ergonomic engineering by analyzing the effects of MSDs on work ability. We found that overexertion at work is highly prevalent, especially when moving patients, and that improper postures and improper force exertion increased MSD incidence among the nursing staff. Other studies have confirmed the correlation between physically demanding work and MSD incidence [53,54]. Regardless of department, most MSDs were observed in the neck, shoulders, and lower back or waist of the nursing staff. Furthermore, medium and high workloads for nurses was found to increase the risk of MSD conditions, which in turn affected their work ability. Based on the conclusions of this study, hospitals and institutions are highly recommended to promote body strengthening and rehabilitation programs for nursing staff.

## Figures and Tables

**Table 1:** Health and lifestyle characteristics of nursing staff.

Variable		Emergency Department Mean (SD)	Intensive Care Unit Mean (SD)	General Ward Mean (SD)
Gender	Male	1 (4%)	1 (2.1%)	0 (0%)
	Female	24 (96%)	45 (97.9%)	46 (100%)
Pregnancy history (female only)	No	18 (75%)	32 (71.1%)	32 (69.6%)
	Yes	6 (25%)	13 (28.9%)	14 (30.4%)
Age		30.7 (3.4)	31.2 (4.5)	30.8 (4.1)
Height		162.5 (5.0)	160 (4.6)	160.7 (5.4)
Weight		60.2 (9.4)	59.1 (13.5)	60.9 (12.4)
Frequency of exercise	Rarely	10 (40)	16 (34.8)	21 (45.7)
	Sometimes	12 (48)	23 (50)	25 (54.3)
	At least once a week	3 (12)	7 (15.2)	0 (0)
Tobacco use	Yes	0 (0%)	1 (2.1%)	0 (0%)
	No	25 (100%)	45 (97.9%)	46 (100%)
Dominant hand	Left	0 (0%)	2 (4.2%)	2 (4.3%)
	Right	25 (10%0)	44 (95.8%)	44 (95.7%)

**Table 2:** Musculoskeletal disorder (MSD) prevalence for each body part in the past year.

Body Part	Emergency Department * (*n* = 25) Number of Occurrences (Prevalence)	Intensive Care Unit * (*n* = 46) Number of Occurrences (Prevalence)	General Wards * (*n* = 46) Number of Occurrences (Prevalence)
Neck	18 (72%)	32 (70%)	37 (80%)
Shoulders	21 (84%)	37 (80%)	37 (80%)
Upper back	9 (36%)	22 (48%)	18 (39%)
Lower back or waist	20 (80%)	36 (78%)	34 (74%)
Elbows	2 (8%)	5 (11%)	7 (15%)
Hands or wrists	7 (28%)	18 (39%)	18 (39%)
Hips or thighs	7 (28%)	16 (35%)	12 (26%)
Knees	9 (36%)	13 (28%)	11 (24%)
Feet and ankles	8 (32%)	15 (33%)	22 (48%)

* *p* < 0.001.

**Table 3:** Hazard ratio analysis between the Nordic musculoskeletal questionnaire (NMQ) and scale I of the checklist for MSDs.

Variable (NMQ)	Musculoskeletal Disorders (MSDs) OR (95% CI)
Elbows	
Low (<19) (*n* = 10)	1.00
Moderate (19–24) (*n* = 77)	1.19 (0.14–10.54)
High (>24) (*n* = 30)	1.39 (0.14–14.07)
Wrists/hands	
Low (<19) (*n* = 10)	1
Moderate (19–24) (*n* = 77)	5.75 (0.69–47.68)
High (>24) (*n* = 30)	6 (0.67–53.68)

Note: OR: odds ratio; CI: confidence interval.

**Table 4:** Hazard ratio analysis between the NMQ and scale II of the checklist for MSDs.

Variable (NMQ)	Musculoskeletal Disorders (MSDs) OR (95% CI)
Upper back	
Low (<10) (*n* = 37)	1.00
Moderate (10–14) (*n* = 31)	0.83 (0.31–2.19)
High (>14) (*n* = 49)	0.98 (0.42–2.33)
Lower back	
Low (<10) (*n* = 37)	1.00
Moderate (10–14) (*n* = 31)	1.86 (0.63–5.46)
High (>14) (*n* = 49)	3.25 (1.14–9.26)
Hips/thighs	
Low (<10) (*n* = 37)	1.00
Moderate (10–14) (*n* = 31)	0.82 (0.28–2.40)
High (>14) (*n* = 49)	1.146 (0.46–2.89)
Knees	
Low (<10) (*n* = 37)	1.00
Moderate (10–14) (*n* = 31)	1.75 (0.57–5.43)
High (>14) (*n* = 49)	2.28 (0.83–6.26)
Ankles/feet	
Low (<10) (*n* = 37)	1.00
Moderate (10–14) (*n* = 31)	0.34 (0.12–0.99)
High (>14) (*n* = 49)	0.88 (0.37–2.08)

Note: OR: odds ratio; CI: confidence interval.

**Table 5:** Hazard ratio analysis between the NMQ and scale III of the checklist for MSDs.

Variable (NMQ)		Musculoskeletal Disorders (MSDs) OR (95% CI)
Rotating the body while handling materials	Lower back	
	No	1.00
	Yes	0.924 (0.35–2.48)
Material handling with one hand	Elbows	
	No	1.00
	Yes	0.579 (0.15–2.21)
	Wrists/hands	
	No	1.00
	Yes	1.14 (0.51–2.56)
Walking more than 3 m while handling/carrying materials	Neck	
	No	1.00
	Yes	3.16 (0.68–14.62)
	Shoulders	
	No	1.00
	Yes	2.03 (0.43–9.54)
	Elbows	
	No	1.00
	Yes	1.60 (0.40–6.42)
	Wrists/hands	
	No	1.00
	Yes	1.11 (0.40–3.13)
	Upper back	
	No	1.00
	Yes	1.13 (0.41–3.11)
	Lower back	
	No	1.00
	Yes	1.60 (0.43–6.00)
	Hips/thighs	
	No	1.00
	Yes	0.89 (0.29–2.70)
	Knees	
	No	1.00
	Yes	0.98 (0.32–3.00)
	Ankles/feet	
	No	1.00
	Yes	1.022 (0.36–2.86)

Note: OR: odds ratio; CI: confidence interval.

**Table 6:** Correlation between the subjective NMQ and the work ability index.

	Time of Symptom Onset	Duration of Symptom	Degree of Impact of Symptom	Frequency of Symptom Occurrence	Work Ability Index
Upper limbs					
Time of symptom onset	1				
Duration of symptom	0.658 **	1			
Degree of impact of symptom	0.764 **	0.598 **	1		
Frequency of symptom occurrence	0.774 **	0.367 **	0.679 **	1	
Work Ability Index	0.256 **	0.223 *	0.237 *	0.144	1
Lower limbs					
Time of symptom onset	1				
Duration of symptom	0.752 **	1			
Degree of impact of symptom	0.770 **	0.746 **	1		
Frequency of symptom occurrence	0.825 **	0.617 **	0.744 **	1	
Work Ability Index	0.307 **	0.287 **	0.264 **	0.287 **	1

* Two-tailed test with a significance level of 0.05; ** Two-tailed test with a significance level of 0.01.

## Data Availability

The data presented in this study are available on request from the corresponding author. The data are not publicly available due to legal restrictions imposed by the government of Taiwan in relation to the “Personal Information Protection Act”.

## Appendix A

**Table A1:** Duration of symptom, type of pain, and impact on life and work for each body part.

Duration of Symptom (Number of People)																			Type of Pain (Number of People)																		Impact on Life and Work (Number of People)																	
	1 Month			3 Months			6 Months			1 Year			3 Years			>3 Years			Soreness			Swelling			Numbness			Tingling			Pain that Wakes One up at Night			Muscle Atrophy			No Impact on Life and Work at All			Slight Reduction in Work Ability			Significant Reduction in Work Ability			Has Required an Absence from Work to Rest			Even Affects One’s Life			Complete Loss of Mobility		
	A	B	C	A	B	C	A	B	C	A	B	C	A	B	C	A	B	C	A	B	C	A	B	C	A	B	C	A	B	C	A	B	C	A	B	C	A	B	C	A	B	C	A	B	C	A	B	C	A	B	C	A	B	C
Neck	7	15	12	2	4	5	2	4	3	1	3	9	1	1	2	5	9	7	18	36	38	0	1	0	3	3	5	0	2	2	0	3	0	0	1	0	9	14	17	8	20	24	0	0	0	0	0	0	0	2	0	0	0	0
Left shoulder	4	20	10	3	1	6	2	4	6	3	2	6	1	0	4	6	11	3	19	37	35	0	0	0	2	6	3	0	4	0	0	1	1	0	1	0	11	15	11	6	20	21	1	1	3	0	0	0	1	2	0	0	0	0
Right shoulder	6	18	8	3	1	6	1	5	4	3	1	6	1	0	3	7	10	7	21	34	36	0	0	0	1	5	3	0	4	0	1	1	1	0	0	0	12	15	13	7	18	20	0	1	3	0	0	0	2	1	0	0	0	0
Upper back	2	11	7	2	2	2	3	5	1	0	2	5	1	0	2	1	5	3	9	24	20	0	0	0	0	2	5	0	2	0	1	0	0	0	0	0	5	6	8	3	16	9	0	0	3	0	0	0	1	3	0	0	0	0
Lower back or waist	4	13	8	2	5	3	5	6	5	4	4	8	1	0	2	5	10	9	20	37	36	0	0	0	3	1	6	2	2	5	2	1	5	0	0	0	11	7	10	7	22	21	1	3	4	0	0	0	2	4	0	0	1	0
Left elbow	1	4	4	1	0	1	0	0	0	0	2	0	0	0	0	0	0	2	2	5	4	0	0	0	0	1	2	0	0	1	0	1	0	0	0	0	1	3	0	1	2	5	0	0	0	0	0	0	0	0	0	0	0	0
Right elbow	1	3	4	1	0	1	0	0	0	0	2	0	0	0	0	0	0	0	2	6	6	0	0	0	0	2	3	0	2	1	0	1	0	0	0	0	1	3	1	1	2	6	0	0	0	0	0	0	0	1	0	0	0	0
Left wrist	2	14	5	0	0	2	0	0	1	1	2	0	0	1	2	0	1	2	6	15	14	0	0	0	4	5	5	1	3	3	0	3	0	0	0	0	2	3	3	1	14	7	0	0	2	0	0	0	0	1	0	0	0	0
Right wrist	2	13	6	1	2	3	0	0	1	3	1	1	0	1	1	0	1	4	6	15	14	0	0	0	4	5	5	1	3	3	0	3	0	0	0	0	2	3	4	3	13	10	1	0	2	0	0	0	0	2	0	0	0	0
Left hip or thigh	2	6	3	0	3	1	2	0	2	0	1	0	0	1	1	2	3	6	5	14	13	0	0	0	2	5	2	0	1	1	0	0	0	0	0	0	4	5	3	1	8	9	1	0	1	0	0	0	0	1	0	0	0	0
Right hip or thigh	3	6	3	0	3	1	2	0	2	0	1	1	0	1	1	2	3	6	6	13	13	0	0	1	2	4	2	0	2	2	0	1	0	0	0	0	4	5	3	1	9	9	1	0	1	0	0	0	0	0	1	0	0	0
Left knee	2	6	3	2	3	3	0	2	0	1	1	0	1	0	0	2	2	6	8	11	11	0	0	0	1	4	2	0	3	1	1	0	2	0	0	1	4	2	3	3	9	8	0	2	1	0	0	0	1	0	0	0	0	0
Right knee	2	4	3	2	3	3	1	3	0	1	0	0	1	0	0	2	1	7	9	10	11	0	0	0	1	3	4	0	1	1	1	0	1	0	0	0	5	2	3	3	7	9	0	2	1	0	0	0	1	0	0	0	0	0
Left foot and ankle	1	5	5	2	1	1	0	1	2	0	3	0	0	0	4	2	2	9	5	10	21	0	3	0	1	2	3	0	1	1	1	0	0	0	0	0	2	1	6	1	10	14	1	1	0	0	0	0	1	0	1	0	0	0
Right foot and ankle	1	6	5	3	1	2	0	1	1	1	3	0	0	0	4	2	2	10	7	13	22	0	2	0	1	1	4	0	0	1	1	0	0	0	0	0	2	3	6	3	10	15	1	0	0	0	0	0	1	0	1	0	0	0

A = emergency department; B = intensive care unit; C = general wards.
